# Magnetocardiographic Rotation Score and Arrhythmic Endpoints in Primary-Prevention ICD Recipients: An Exploratory Substudy of the Magneto-SCD Trial

**DOI:** 10.3390/jcm15186985

**Published:** 2026-09-09

**Authors:** Thomas Lachlan, Hejie He, Adam Miller, Nakul Chandan, Taesoon Hwang, Shoaib Siddiqui, Roger Beadle, David Wilson, Sanjiv Petkar, Ven Gee Lim, Peter K. Kimani, Faizel Osman

**Affiliations:** 1 Institute for Cardio-Metabolic Medicine, University Hospitals Coventry and Warwickshire NHS Trust, Coventry CV2 2DX, UK; thomas.lachlan@uhcw.nhs.uk (T.L.); nakul.chandan@uhcw.nhs.uk (N.C.); taesoon.hwang@uhcw.nhs.uk (T.H.); vengee.lim@uhcw.nhs.uk (V.G.L.); 2 Department of Life Sciences, Warwick Medical School, University of Warwick, Coventry CV4 7AL, UK; peter.k-u.kimani@warwick.ac.uk; 3 School of Physics and Astronomy, University of Leeds, Leeds LS2 9JT, UK; adammorrisonmiller@gmail.com; 4 Centre for Health and Life Sciences, Coventry University, Coventry CV1 5FB, UK; 5 Department of Cardiology, George Eliot Hospital, Nuneaton CV10 7DJ, UK; shoaib.siddique@swft.nhs.uk; 6 Department of Cardiology, Warwick Hospital, South Warwickshire University NHS Foundation Trust, Warwick CV34 5BW, UK; roger.beadle@swft.nhs.uk; 7 Department of Cardiology, Worcester Royal Hospital, Worcester WR5 1DD, UK; david.wilson45@nhs.net; 8 Department of Cardiology, Wolverhampton Heart Centre, Royal Wolverhampton NHS Trust, Wolverhampton WV10 0QP, UK; sanjiv.petkar@nhs.net

**Keywords:** magnetocardiography, implantable cardioverter-defibrillator, sudden cardiac death, ventricular arrhythmia, primary prevention, MADIT-ICD benefit score, competing risk, arrhythmogenic substrate, non-invasive electrophysiology

## Abstract

**Background/Objectives:** Primary-prevention implantable cardioverter-defibrillator (ICD) selection remains dominated by left ventricular ejection fraction and clinical heart failure markers, which incompletely distinguish between patients who will experience ventricular arrhythmia and those whose prognosis is limited by competing non-arrhythmic mortality. The Magneto-SCD trial recently reported novel rotation-based magnetocardiography (MCG) indices associated with future appropriate ICD therapies. This substudy explores whether MCG Rotation Score provides incremental information in primary-prevention ICD recipients, particularly beyond NYHA status and MADIT-arrhythmic and non-arrhythmic risk scores. **Methods:** We included all Magneto-SCD trial participants with a primary-prevention indication with non-missing follow-up/MCG Rotation Score data. The full primary-prevention cohort comprised 54 participants; the post-MI subgroup comprised 28. The primary endpoint was time to first appropriate ICD therapy. Death without prior therapy was used as a pragmatic competing endpoint and not assumed to represent adjudicated non-arrhythmic death. Appropriate shock was a secondary endpoint. Two-year cumulative incidence functions were estimated using Aalen–Johansen methods. Cause-specific Cox models assessed Rotation Score alone and after addition to MADIT-derived scores; nested likelihood-ratio tests evaluated model fit. **Results:** Of 104 participants, 90 had analysable MCG data and 65 had a primary-prevention ICD indication; after exclusions for missing patients, 54 patients remained (mean age 63.8 ± 13.2 yrs, 45(83.3%) male). Appropriate ICD therapies occurred in 10 (18.5%), including appropriate ICD shock in eight (14.8%); 10 patients (18.5%) died without prior therapy. The median follow-up was 1211.5 days (IQR 793.5–1396.0). Each SD increase in Rotation Score was associated with appropriate ICD therapy with HR 1.64 (95% CI 0.93–2.87; *p* = 0.086), appropriate ICD shock with HR 2.07 (95% CI 1.15–3.72; *p* = 0.015), death without prior therapy with HR 1.08 (95% CI 0.54–2.17; *p* = 0.824), and death without prior ICD shock with HR 0.95 (95% CI 0.48–1.90; *p* = 0.890). Adding Rotation Score did not significantly improve the primary therapy model beyond MADIT components (LRT *p* = 0.065) or MADIT benefit score (LRT *p* = 0.109). Model fit improved for the secondary appropriate ICD shock endpoint when Rotation Score was added to MADIT components (LRT *p* = 0.016), based on eight shock events. **Conclusions:** Rotation Score showed an association pattern more closely aligned with arrhythmic endpoints, particularly appropriate ICD shock, than with death without prior therapy. Incremental prognostic value for the primary endpoint was not established. These findings are hypothesis-generating and require confirmation in adequately powered, prospectively designed and externally validated cohorts before any role in clinical risk stratification or ICD decision-making can be determined.

## 1. Introduction

Sudden cardiac death (SCD) is a major public health problem and is commonly mediated by ventricular tachyarrhythmias (VA) in patients with structural heart disease [1,2]. Implantable cardioverter-defibrillators (ICDs) reduce arrhythmic death in selected high-risk populations, but contemporary primary-prevention candidates selection remains imperfect [2,3,4,5]. Left ventricular ejection fraction (LVEF) is clinically important, yet it does not directly measure the electrophysiological substrate supporting sustained re-entry or ventricular fibrillation.

**Figure 1:**
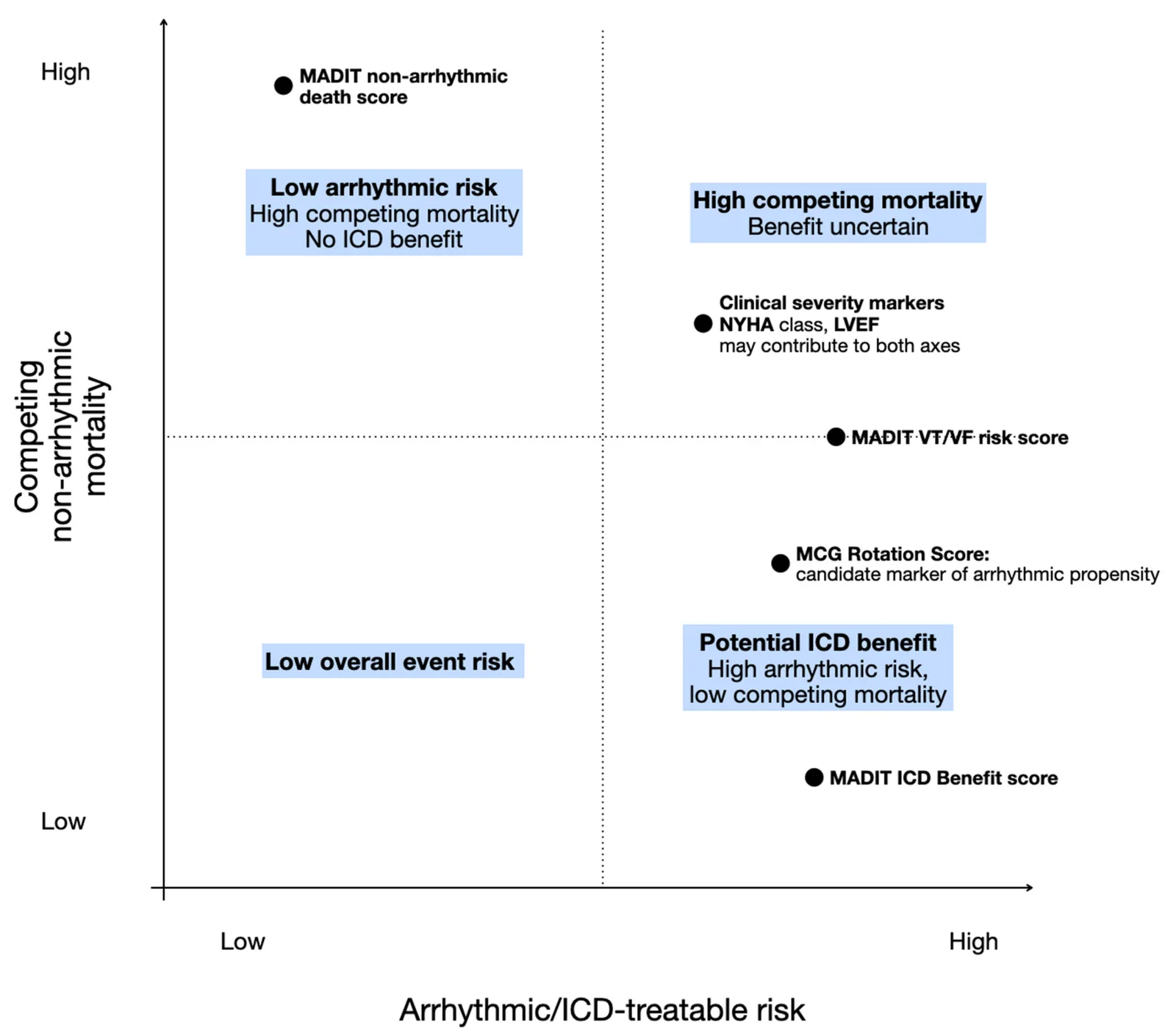
Proposed two-axis framework for ICD risk stratification. MCG Rotation Score is hypothesised to contribute preferentially to the arrhythmic/therapy-risk axis, whereas clinical severity markers such as NYHA class and LVEF may contribute to both arrhythmic and competing-mortality risk. The MADIT non-arrhythmic death score is expected to align more strongly with the competing-mortality axis. Potential ICD benefit is greatest when arrhythmic risk is high and competing non-arrhythmic mortality is low.

ICD benefit depends on the consideration of ICD-treatable arrhythmic risk and the risk of death from non-arrhythmic mechanisms. The MADIT-ICD benefit score formalised these domains by combining a ventricular tachycardia/ventricular fibrillation (VT/VF) risk score with a non-arrhythmic death score [6]. Figure 1 presents this established two-axis framework; the possible contribution of MCG Rotation Score is shown only as the study hypothesis tested subsequently, not as an observed result or calibrated clinical position. Contemporary reassessment of primary-prevention ICD selection further highlights the limitations of LVEF-only selection [7,8].

Magnetocardiography (MCG) is a contactless technique that records the biomagnetic field generated by cardiac electrical activity. Magnetic fields are less distorted by intervening tissue conductivity than surface voltage measurements; MCG may preserve electrophysiological information that is attenuated or spatially blurred on the body-surface electrocardiogram (ECG) as well as represent tangential components and electrical vortex currents [9,10]. Historically, clinical MCG was constrained by cryogenic sensors and magnetically shielded rooms. The Magneto-SCD trial utilised a portable, room-temperature, unshielded MCG system, enabling viable acquisition in routine clinical environments [11,12].

The Magneto-SCD trial evaluated established and novel MCG parameters in de novo ICD and cardiac resynchronisation therapy defibrillator (CRT-D) recipients. Two novel MCG parameters (Rotation Score and Angular Dynamics) were developed that quantify temporal re-orientation of the dominant MCG dipole during ventricular depolarisation [13]. In the main trial, rotation-based indices were associated with subsequent appropriate ICD therapies, particularly shocks, suggesting that they may capture conduction heterogeneity or an arrhythmogenic substrate not fully reflected by LVEF or conventional clinical markers [14]. The clinical value of such a marker would be greatest in the primary-prevention setting, where the patient has not yet declared arrhythmic risk through prior sustained VA. To limit multiplicity and model complexity in this sparse-event substudy, Rotation Score was selected as the single representative MCG exposure. We examined whether it was associated preferentially with arrhythmic endpoints and whether it showed possible information beyond MADIT-derived risk constructs. The post-MI subgroup was included for conceptual comparison with contemporary PROFID-EHRA questions.

## 2. Materials and Methods

### 2.1. Study Design and Population

This was a secondary analysis of the prospective, multicentre, observational Magneto-SCD study [11,12,13,14], which recruited adults undergoing de novo ICD or CRT-D implantation at five UK hospitals between September 2019 and November 2021. ICD or CRT-D selection was made by the treating team according to clinical indications for defibrillation and cardiac resynchronisation; device type was not randomised by the study. The present analysis was restricted to primary-prevention indications and with recorded follow-up and a non-missing Rotation Score modelling. The trial was registered with ClinicalTrials.gov (NCT04352816). The study was approved by the York and Humber Regional Ethics Committee (19/YH/0143; approval date: 17 July 2019).

Two analysis groups were prespecified: The full primary-prevention cohort included all eligible ICD/CRT-D recipients. The post-MI subgroup additionally required documented prior MI at least 3 months prior to entry into the main study. The full cohort addressed the general primary-prevention question; the post-MI subgroup provided only a descriptive, conceptually relevant comparison with the population being assessed in the contemporary PROFID-EHRA study, while recognising that the present dataset was not designed as a PROFID replication cohort [8].

### 2.2. Magnetocardiography Acquisition and Rotation Score

MCG acquisition and processing followed the Magneto-SCD protocol [11]. Recordings were obtained using a portable, room-temperature, unshielded device (VitalScan, Creavo Medical Technologies, Coventry, UK). Recordings were performed with participants in the supine position in routine hospital clinical settings. Data underwent quality assessment, including signal-to-noise review and visual inspection; recordings with excessive environmental interference or an unclear cardiac signal were excluded from the parent analysable MCG cohort.

During the QRS interval, sequential magnetic field maps were generated. At each timepoint, the positive and negative pole centroids were identified, and the dominant dipole orientation was defined from the negative to the positive pole. Rotation Score was calculated as the cumulative absolute change in dipole angle between consecutive timepoints, weighted by instantaneous MCG amplitude to reduce the influence of very low-amplitude frames [13]. Higher values therefore indicate greater temporal re-orientation of the dominant magnetic dipole during ventricular depolarisation. Rotation Score was analysed continuously and hazard ratios (HR) were expressed per standard deviation (SD) increase. Rotation Score was selected as the primary MCG exposure because its performance was broadly similar to Angular Dynamics in the parent analysis, while reducing multiplicity and over-parameterisation in this small-event substudy.

### 2.3. Comparator Variables and Risk Constructs

Clinical covariates included age, LVEF, NYHA class, and ischaemic aetiology. NYHA status was reconstructed retrospectively from clinical documentation and dichotomised as NYHA < 2 versus NYHA ≥ 2 because exact separation of class II from class III was not reliable for all participants. In this dataset, NYHA ≥ 2 therefore represented documented class II or III; no NYHA IV participant was identified in the cohort (in keeping with current guidelines). LVEF was treated as a percentage. NYHA class was dichotomised as NYHA >2 versus NYHA < 2. MADIT-derived scores available in the dataset were used as comparator constructs: the VT/VF score, the non-arrhythmic death score, and the ICD benefit score. For modelling, the ICD-benefit score was scaled as benefit10 = benefit score/10. The principal incremental-value question was whether Rotation Score added information beyond the MADIT-derived arrhythmic and competing-mortality component scores.

### 2.4. Endpoints

The primary endpoint was time to first appropriate ICD therapy, defined as anti-tachycardia pacing (ATP) or ICD shock, judged appropriate according to device interrogation and clinical adjudication in the parent study. Participants without appropriate therapy were followed to death or last recorded follow-up. Death without prior therapy was treated as a pragmatic competing endpoint; it was not classified as adjudicated non-arrhythmic death. For participants who died without prior therapy, event time was the recorded interval derived from implant and death dates. Participants alive without therapy at last follow-up were censored.

A secondary endpoint examined appropriate ICD shock as a more specific but less frequent arrhythmic proxy than any device therapy. ICD shock events required an appropriate ICD shock status and a valid time from implant to appropriate ICD shock. Death without prior shock was handled analogously to the primary analysis as a competing event.

### 2.5. Statistical Analysis

Details of the statistics used in the Magneto-SCD trial have been reported [11,14]. Descriptive analyses reported cohort derivation, variable-specific missingness, median follow-up with interquartile range and maximum follow-up, appropriate ICD therapy, appropriate ICD shock, ATP and competing-event counts. Aalen–Johansen cumulative incidence functions (CIFs) were estimated for appropriate ICD therapy and death without prior therapy and summarised at two years (730 days). Exploratory strata were cohort-specific rank-based tertiles of Rotation Score and MADIT-derived scores in the full cohort and Rotation Score tertiles and NYHA < 2 versus NYHA ≥ 2 in the post-MI subgroup.

Cause-specific Cox models were fitted separately for the event of interest and the competing event. Cause-specific hazards were selected because the principal objective was to examine aetiological associations with the instantaneous event rate among participants who were still event-free; Aalen–Johansen methods were used to present absolute cumulative incidence. Fine-Gray subdistribution models were not used as the primary regression approach because this small exploratory analysis was not designed to estimate a treatment-decision subdistribution effect [15]. Rotation Score was modelled per SD on a linear scale; restricted cubic splines were not fitted because only 8–10 events were available for the principal models. Proportional-hazards assumptions were assessed using scaled Schoenfeld residuals.

Prespecified full-cohort models included Rotation Score alone, MADIT benefit score with and without Rotation Score, and the MADIT VT/VF and non-arrhythmic death component scores with and without Rotation Score. Clinical multivariable models were considered exploratory and were not emphasised when unstable. Nested likelihood-ratio tests compared models with and without Rotation Score. Apparent C-index and two-year calibration were explored using 500 bootstrap resamples, but NRI, IDI and decision-curve analysis were not undertaken because of sparse events and the absence of an externally validated clinical threshold.

Analyses were performed in RStudio (2025.09.2+418; RStudio Inc., Boston, MA, USA) using the survival package for cause-specific Cox models and custom R functions for Aalen–Johansen cumulative-incidence estimation and bootstrap validation.

## 3. Results

### 3.1. Cohort Outcomes

**Figure 2:**
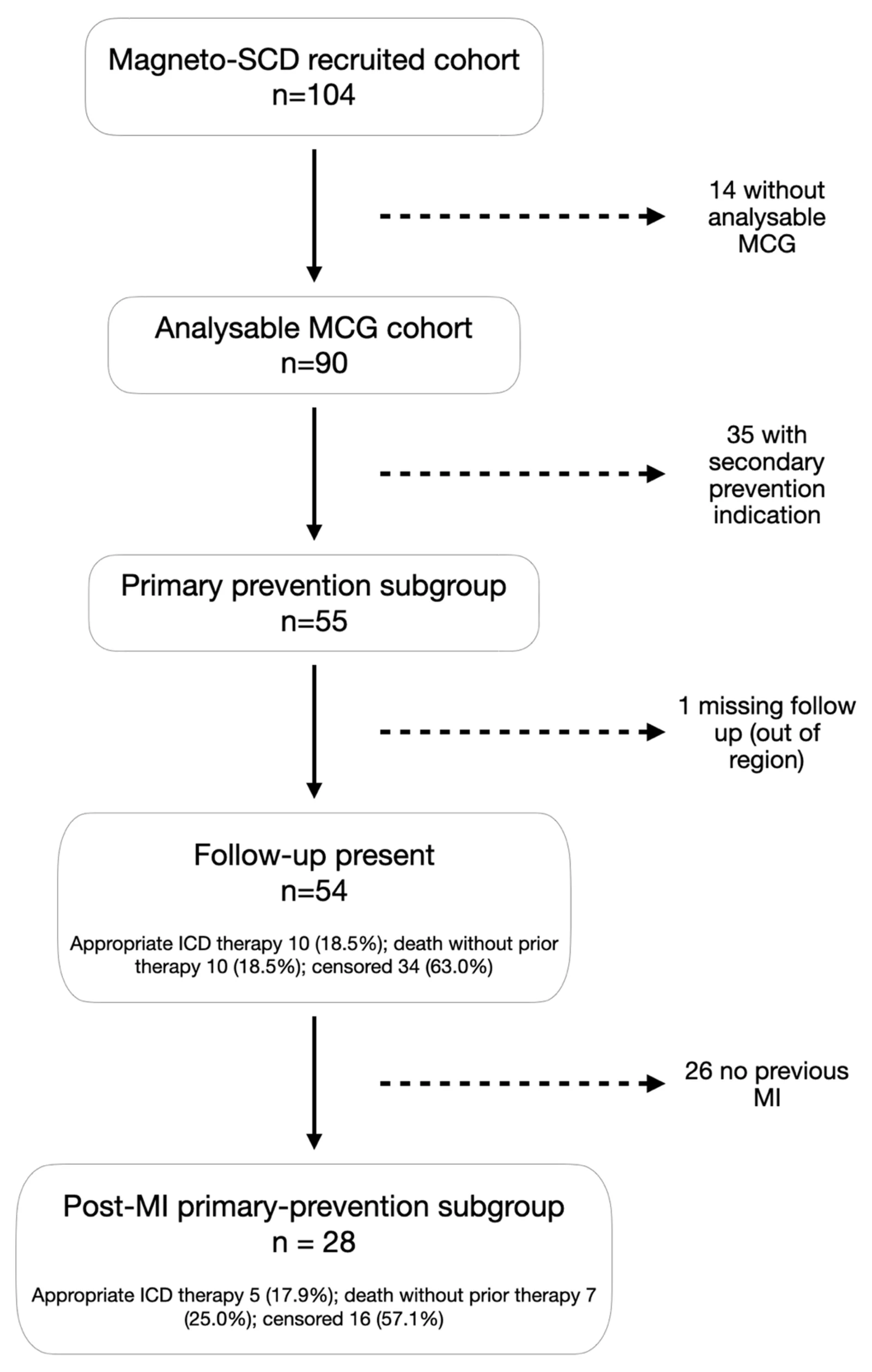
Study flow diagram. Flow from the recruited Magneto-SCD cohort to the analysable MCG cohort, primary-prevention cohort, Cohort A, and prior-MI Cohort B, with event accounting for appropriate ICD therapy, death without prior therapy, and censoring.

The Magneto-SCD study recruited 104 participants, of whom 90 had analysable MCG data [14]. Sixty-five participants in the recruited cohort had a primary-prevention indication. After restriction to participants with non-missing Rotation Score data and required risk-stratification variables, 54 primary-prevention ICD/CRT-D recipients were identified; mean age was 63.8 ± 13.2 yrs and 45 (83.3%) were males. Of these, 10 (18.5%) experienced appropriate ICD therapy, 10 (18.5%) died without prior therapy, and 34 (63.0%) were censored alive without prior therapy. The post-MI subgroup included 28 participants, of whom five (17.9%) experienced appropriate ICD therapy, seven (25.0%) died without prior therapy, and 16 (57.1%) were censored. Median follow-up was 1211.5 days (IQR 793.5–1396.0; maximum 2069) in the full cohort and 1256.0 days (IQR 822.8–1489.5; maximum 2069) in the post-MI subgroup. The cohort derivation and event accounting are summarised in Figure 2. Baseline patient characteristics are reported in Table S1.

### 3.2. Two-Year Competing-Risk Estimates

**Figure 3:**
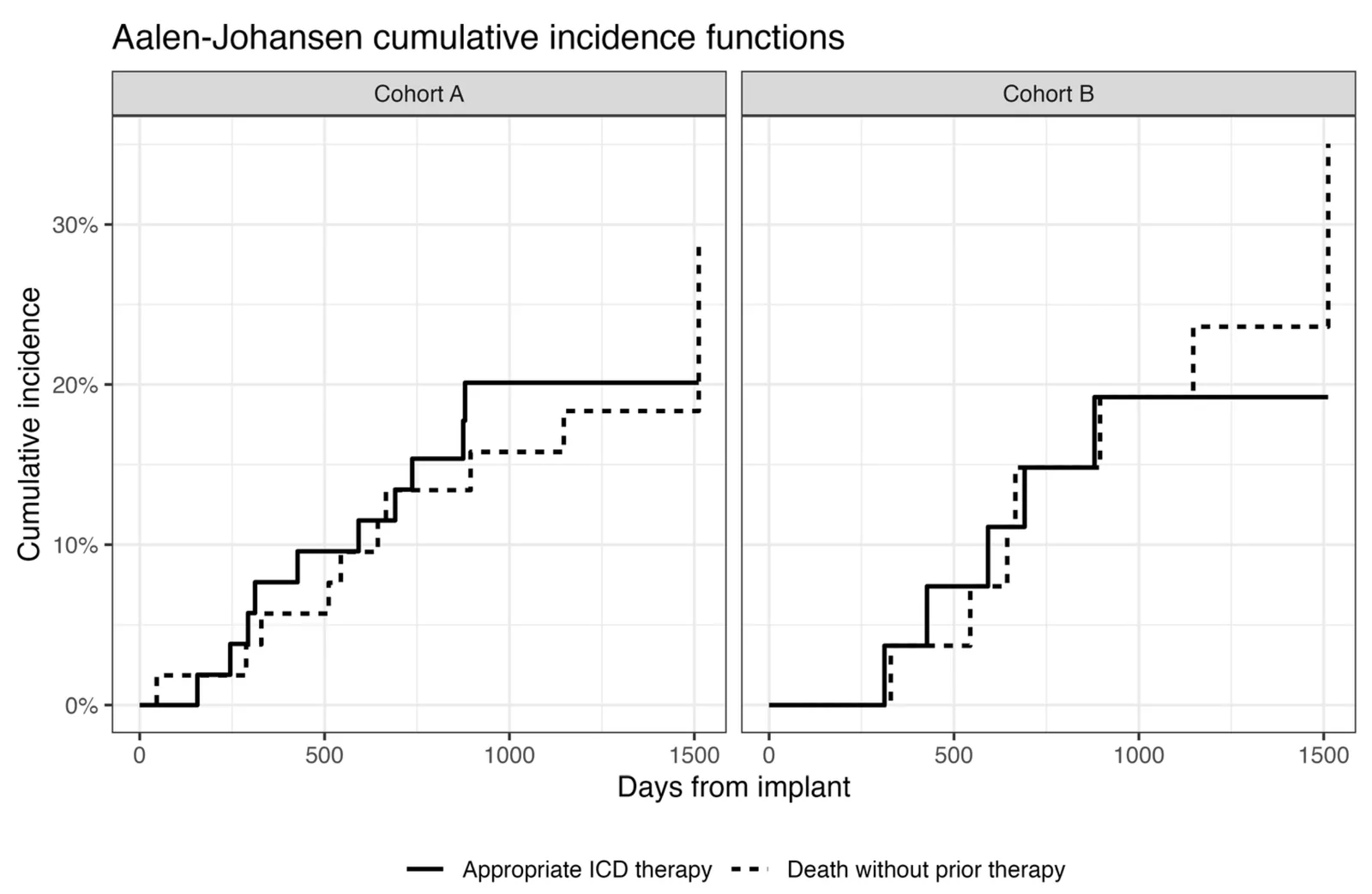
Aalen–Johansen cumulative incidence functions for appropriate ICD therapy and death without prior therapy. Curves show competing-risk estimates for Cohort A and Cohort B, with death without prior therapy treated as a competing event for appropriate ICD therapy.

At two years, the cumulative incidences of appropriate ICD therapy and death without prior therapy were each 0.134 in the full primary-prevention cohort, with event-free survival 0.732 (Figure 3; Table 1). Corresponding CIF estimates in the post-MI subgroup were each approximately 0.148, with event-free survival of approximately 0.704. In the five post-MI participants with NYHA < 2, no endpoint occurred by two years (Table S5). Given the very small NYHA < 2 denominator and a later therapy event in that group, this was treated as a descriptive observation that may reflect chance rather than evidence of reliable risk separation.

### 3.3. NYHA Status, Rotation Score and Post-MI Competing Risk

In the post-MI subgroup, NYHA status and Rotation Score showed different endpoint patterns. All seven deaths without prior therapy among participants with available NYHA classification occurred in those with NYHA ≥ 2, but the NYHA coefficient was not reliably estimable because the NYHA < 2 stratum contained no such deaths. Over complete follow-up, one NYHA < 2 participant experienced appropriate ICD therapy and shock. When Rotation Score was split at the post-MI median (391.1) for description only, all four appropriate ICD shocks occurred in the above-median group, whereas deaths without prior therapy occurred in both Rotation groups (Tables S2 and S5). These subgroup patterns are hypothesis-generating and are not validated thresholds.

### 3.4. Rotation Score and Cause-Specific Risk of Therapy Versus Competing Death

**Figure 4:**
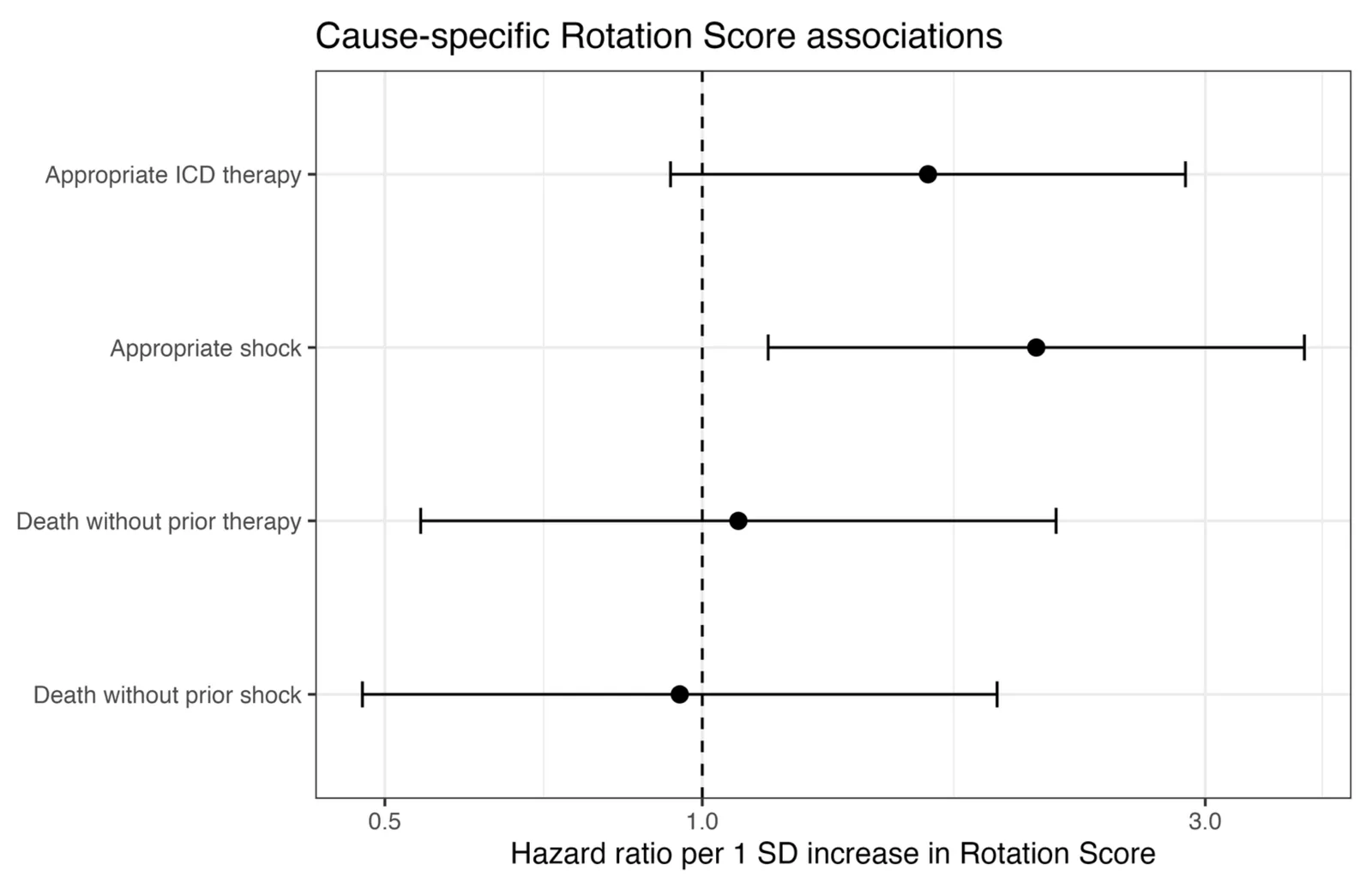
Cause-specific hazard ratios for Rotation Score in Cohort A. Hazard ratios are shown per 1 SD increase in Rotation Score for appropriate ICD therapy, appropriate shock, death without prior therapy, and death without prior shock. Models are cause-specific Cox models.

In the full primary-prevention cohort, each SD increase in Rotation Score was associated with a higher hazard of appropriate ICD therapy, although this did not reach statistical significance (HR 1.64, 95% CI 0.93–2.87; *p* = 0.086). The association was stronger when appropriate shock was used as the arrhythmic endpoint, HR 2.07, 95% CI 1.15–3.72. Corresponding associations were close to null for death without prior therapy (HR 1.08, 95% CI 0.54–2.17; *p* = 0.824) and death without prior ICD shock (HR 0.95, 95% CI 0.48–1.90; *p* = 0.890) (Table 2; Figure 4). Schoenfeld residual tests provided no evidence of proportional-hazards violations in the four Rotation-only models or the corresponding MADIT-components-plus-Rotation models, although the diagnostics had limited power because of sparse events (all global or relevant term-specific *p* ≥ 0.504; Table S6).

### 3.5. Rotation Score Added to MADIT-Derived Risk Constructs

When Rotation Score was added to the VT/VF score and non-arrhythmic death score component model for appropriate ICD therapy, its coefficient was HR 1.80, 95% CI 1.01–3.21 (Table 3). However, the nested likelihood-ratio comparison did not meet the threshold for improved primary-endpoint model fit (LRT *p* = 0.065). The comparison with the MADIT benefit score was also non-significant (LRT *p* = 0.109). For death-without-therapy, adding Rotation Score did not improve model fit beyond MADIT components (LRT *p* = 0.790) or MADIT benefit score (LRT *p* = 0.992) (Table 3).

For the secondary appropriate ICD shock endpoint, Rotation Score in the MADIT-component model had HR 2.25 (95% CI 1.21–4.16), and the nested model comparison was statistically significant (LRT *p* = 0.016). No model-fit difference was observed for death without prior ICD shock (Rotation HR 0.97, 95% CI 0.48–2.00; LRT *p* = 0.942). Because these analyses were based on only eight shock events, they may be sensitive to influential observations and do not establish clinical incremental value. (Table 3 and Table S3).

### 3.6. Two-Year Prediction Modelling

Exploratory two-year prediction models showed numerically higher apparent and optimism-corrected discrimination for some MADIT models containing Rotation Score (Table S4). However, event counts were low and several fitted models showed convergence warnings or calibration instability and bootstrap optimism correction could not compensate for inadequate event numbers. Prediction metrics were therefore retained as supplementary hypothesis-generating analyses rather than evidence of a clinically usable prediction model.

## 4. Discussion

### 4.1. Principal Findings

This exploratory substudy identified an association pattern in which Rotation Score was more strongly related to appropriate ICD shock than to the broader appropriate ICD therapy endpoint, while showing no association with death without prior therapy or death without prior ICD shock. The primary-endpoint likelihood-ratio comparisons beyond MADIT-derived models were non-significant; only the secondary appropriate ICD shock comparison reached statistical significance. Accordingly, the findings support a possible arrhythmia-related signal but do not establish incremental prediction, clinical utility, or a role in ICD selection.

These findings are conceptually important because they suggest that Rotation Score may not simply be a marker of advanced global illness, heart failure severity, or frailty. A marker that increases both therapy risk and competing death risk, such as LVEF, may not aid ICD selection, because high arrhythmic risk may be offset by high non-arrhythmic mortality. By contrast, a marker preferentially associated with ICD-treatable VA could complement clinical scores designed to balance arrhythmic and non-arrhythmic risk. In this exploratory dataset, Rotation Score appeared to have its greatest effect on the arrhythmic/therapy axis. Importantly, death without prior therapy is only a pragmatic competing endpoint and cannot be equated with adjudicated non-arrhythmic death. Appropriate ICD therapy is also an imperfect surrogate for sudden death prevented because programming, ATP and spontaneous arrhythmia termination influence whether an episode is recorded and treated. Real-world remote-monitoring literature similarly illustrates the dependence of device-detected events on surveillance and programming [16].

### 4.2. Relationship to MADIT, PROFID-EHRA and CONTEMP-ICD Frameworks

The MADIT-ICD benefit score separates predicted VT/VF risk from predicted non-arrhythmic mortality risk, recognising that ICD benefit is greatest when arrhythmic risk is high and competing mortality is low [6]. The present analysis used this logic not to replace MADIT-derived scores, but to test whether a contactless MCG biomarker might add substrate information to the arrhythmic side of the equation. The absence of a Rotation Score signal for death without therapy is notable because it suggests that Rotation Score may measure a different biological domain than clinical variables such as age, diabetes, NYHA class, renal dysfunction, or body habitus, which often reflect competing mortality burden.

The post-MI subgroup was included because PROFID-EHRA is reassessing prophylactic ICD implantation after MI under contemporary therapy [8]. A central premise of this framework is that symptomatic heart-failure severity, reflected by NYHA ≥ 2 status, may identify patients with higher competing non-arrhythmic mortality, in whom the incremental benefit of ICD implantation may be attenuated because a greater proportion of adverse outcomes may not be preventable by defibrillation. The present subgroup was far smaller, NYHA status was reconstructed retrospectively and only five therapy events occurred. Its NYHA findings should therefore be regarded as descriptive and potentially attributable to chance, not as replication, validation or evidence for withholding or implanting an ICD.

The CONTEMP-ICD concept similarly reflects the need to re-evaluate primary-prevention ICD therapy under contemporary heart failure treatment, especially among patients with lower predicted arrhythmic risk relative to non-arrhythmic mortality [7]. The present two-axis analysis is therefore aligned with the direction of the field: future ICD selection will likely require markers that clarify which patients are at highest risk of shockable arrhythmia, which patients are primarily at risk of non-arrhythmic death, and which patients have a favourable balance for ICD implantation.

MCG should also be considered within the wider landscape of multimodality sudden-death risk assessment rather than only against LVEF and MADIT scores. Candidate approaches include CMR late gadolinium enhancement, scar burden and heterogeneity, global longitudinal strain, ECG markers such as QRS fragmentation and T-wave alternans, biomarkers, and programmed ventricular stimulation in selected populations. PRESERVE EF illustrates a combined non-invasive and electrophysiological strategy after MI [17], while DANISH and CMR studies emphasise the limitations of relying on LVEF alone [18,19,20].

### 4.3. Biological Plausibility

Rotation Score quantifies cumulative re-orientation of the dominant magnetic dipole during ventricular depolarisation [13]. A stable, homogeneous activation sequence would be expected to produce a more consistent dipole trajectory, whereas scar, fibrosis, conduction slowing, wavefront fragmentation, or functional block could plausibly generate greater temporal re-orientation. In patients with cardiomyopathy or prior MI, such abnormalities may reflect channels and barriers capable of supporting re-entry. The stronger association with appropriate ICD shock is physiologically compatible with this hypothesis. This interpretation remains inferential, particularly in the context of sparse events, programming differences or influential observations. The study did not include systematic scar imaging, electroanatomic mapping, or invasive electrophysiological validation. Future studies should determine whether Rotation Score correlates with late gadolinium enhancement burden, scar border-zone heterogeneity, conduction-channel metrics, ventricular electrogram fractionation, or inducibility. Such mechanistic validation would help establish whether Rotation Score is a direct marker of substrate complexity or an indirect correlate of other electrophysiological or structural abnormalities.

### 4.4. Clinical Implications

No Rotation Score threshold can currently be recommended, and no current data can establish that withholding an ICD at a low value would be safe. Before clinical application, Rotation Score would need reproducibility testing, external validation, calibration, discrimination beyond established markers, reclassification and decision-curve analyses, and evidence that its use changes decisions or outcomes. If externally validated, the MCG Rotation Score could have several potential clinical applications. First, it could support shared decision-making in borderline primary-prevention ICD candidates by providing a non-invasive measure of VA risk propensity. Second, it could complement MADIT-derived or other competing-risk models by supplying electrophysiological substrate information rather than only clinical comorbidity data. Third, it could help refine risk stratification in post-MI and cardiomyopathy populations undergoing reassessment in contemporary trials. The potential role of MCG may also be studied within temporary protection strategies using a wearable cardioverter-defibrillator (WCD). In recent MI, newly diagnosed cardiomyopathy, myocarditis or another potentially reversible substrate, arrhythmic risk may be transient and permanent ICD implantation may be premature. A WCD can provide temporary protection while guideline-directed therapy, ventricular function and long-term candidacy are reassessed. Future studies could evaluate Rotation Score alongside WCD-derived arrhythmic and adherence data, imaging, biomarkers and clinical variables [21,22]. Finally, because the parent Magneto-SCD study [14] used a portable unshielded device, the workflow may be more compatible with routine hospital practice than earlier shielded-room MCG systems.

The potential clinical utility of improved arrhythmic risk stratification should also be considered in relation to the procedural and long-term risks of ICD implantation. Cardiac implantable electronic device infection is among the most serious complications and may require complete system extraction, prolonged antibiotic therapy and delayed reimplantation. Therefore, the decision to implant an ICD should balance not only arrhythmic risk and competing mortality, but also patient-specific procedural and infectious risk. Contemporary prevention relies on careful pre-procedural risk assessment, optimisation of modifiable factors, systemic antibiotic prophylaxis, meticulous sterile technique, prevention of pocket haematoma and selective use of adjunctive antibacterial strategies in high-risk patients [23,24].

### 4.5. Strengths

The current study has several strengths. It uses a real-world cohort to focus on a clinically highly relevant primary-prevention ICD population, where risk stratification has the greatest unmet need. It uses time-to-event and competing-risk methods rather than relying solely on binary event status. It evaluates both a broad ICD therapy endpoint and a more specific ICD shock endpoint. It also explicitly tests whether Rotation Score adds to established MADIT-derived constructs and whether any incremental information is endpoint-specific. Finally, the analysis is grounded in a prospective multicentre MCG cohort using a portable device in routine clinical settings.

### 4.6. Limitations

The dominant limitation is event scarcity: the full primary-prevention cohort included only 10 appropriate ICD therapy events, 10 deaths without prior therapy and only eight appropriate ICD shocks; the post-MI subgroup contained five therapy events. Multivariable coefficients, likelihood-ratio tests, subgroup comparisons and bootstrap estimates are consequently unstable and vulnerable to separation, overfitting and influential observations. Several clinical risk-stratification variables were not prespecified in the original Magneto-SCD study design and were reconstructed after trial completion from clinical records.

Endpoint classification also limits interpretation. Appropriate ICD therapy is influenced by detection zones, ATP algorithms, programming changes and adjudication, while appropriate ICD shock is more specific but much less frequent. Programming was not standardised centrally and some ATP occurred below 180 beats/min. Death without prior therapy is not equivalent to non-arrhythmic death: unwitnessed arrhythmic death, terminal device deactivation or incomplete peri-terminal interrogation could lead to misclassification. The revised analysis uses documented implant-to-death timing for all recorded deaths and does not substitute last follow-up as an event date, but cause of death was not centrally adjudicated.

The analysis was performed in a single study cohort without external validation. Several risk variables, particularly NYHA status and MADIT components, were reconstructed retrospectively and contained missing or unclassifiable values. Exact NYHA II versus III separation was unavailable, and the post-MI subgroup was too small for reliable subgroup inference. Recruitment occurred during the COVID-19 era, which may have altered referral patterns, implantation practice, follow-up and competing mortality. The parent study was also under-recruited because of the pandemic, a time-limited CE mark for the device, and non-analysable scans due to environmental magnetic interference, which may have introduced selection bias. Test–retest, operator, site and processing-pipeline reproducibility were not systematically assessed.

### 4.7. Future Directions

Future studies should prospectively validate Rotation Score in adequately powered primary-prevention cohorts with standardised device programming, central adjudication of arrhythmia and cause of death, contemporary therapy documentation and independent external validation. Comparative analyses should evaluate incremental value beyond LVEF, NYHA class, CMR scar characteristics, ECG/Vectorcardiographic markers, biomarkers and competing-risk models such as the MADIT-ICD benefit score. Validation should assess non-linearity, calibration, discrimination, reclassification, decision curves, thresholds and whether the Rotation Score changes treatment recommendations or patient outcomes. Mechanistic studies should link MCG-rotation metrics to structural scar architecture and invasive electrophysiology, while implementation studies should quantify reproducibility, acquisition failure rates and environmental noise effects.

## 5. Conclusions

In this exploratory primary-prevention Magneto-SCD substudy, Rotation Score was associated with appropriate ICD shock and showed a weaker, non-significant association with the broader appropriate ICD therapy endpoint, while showing no association with death without prior therapy. Adding Rotation Score did not significantly improve primary-endpoint model fit beyond the evaluated MADIT-derived models; a model-fit signal was observed only for the secondary shock endpoint and was based on eight events. These findings suggest that Rotation Score may provide information related to ventricular arrhythmic propensity that is not fully captured by the evaluated clinical scores. Larger, prospectively designed and externally validated studies are required to determine whether this association improves clinical risk stratification or ICD decision-making.

## Figures and Tables

**Table 1:** Two-year Aalen–Johansen cumulative incidence estimates.

Cohort	CIF Therapy at 2 Years	CIF Death Without Therapy at 2 Years	Event-Free Survival at 2 Years	Interpretation
A: Primary prevention	0.134	0.134	0.732	Arrhythmic/therapy and competing death risks were numerically similar by two years.
B: Primary prevention + prior MI	0.148	0.148	0.704	Similar competing-risk balance in the post-MI subgroup, with sparse events.

Footnote: CIF estimates were calculated using the Aalen–Johansen method at 730 days. Death without prior therapy was treated as a competing event for appropriate ICD therapy. Event-free survival was calculated as 1 minus the sum of the therapy and death-without-therapy CIFs. CIF, cumulative incidence function; MI, myocardial infarction.

**Table 2:** Cause-specific Cox model summary for Rotation Score in Cohort A.

Endpoint/Model	Rotation Scale	HR	95% CI	*p* Value	Interpretation
Appropriate ICD therapy: Rotation Score only	Per 1 SD increase	1.64	0.93–2.87	0.086	Suggestive association with therapy.
Appropriate ICD shock: Rotation Score only	Per 1 SD increase	2.07	1.15–3.72	0.015	Stronger association with the more specific arrhythmic endpoint.
Death without prior therapy: Rotation Score only	Per 1 SD increase	1.08	0.54–2.17	0.824	No evidence of association with competing death.
Death without prior shock: Rotation Score only	Per 1 SD increase	0.95	0.48–1.90	0.890	No corresponding competing-death signal.

Footnote: Hazard ratios are from cause-specific Cox models and are expressed per 1 SD increase in Rotation Score. For therapy and shock models, competing deaths were censored at the time of death. For death-without-therapy and death-without-shock models, prior therapy or shock events were censored at the event time. CI, confidence interval; ICD, implantable cardioverter-defibrillator; SD, standard deviation.

**Table 3:** Nested model comparisons after adding Rotation Score to MADIT-derived constructs in the full primary-prevention cohort.

Comparison	Endpoint	Rotation Estimate	Likelihood Ratio Test *p* Value	Interpretation
MADIT benefit score + Rotation Score vs. MADIT benefit score	Appropriate ICD therapy	HR 1.65, 95% CI 0.93–2.94	LRT *p* = 0.109	No statistically significant improvement in primary-endpoint model fit.
MADIT benefit score + Rotation Score vs. MADIT benefit score	Death without prior therapy	HR 1.00, 95% CI 0.49–2.06	LRT *p* = 0.992	No statistically significant model-fit improvement.
MADIT components + Rotation Score vs. MADIT components	Appropriate ICD therapy	HR 1.80, 95% CI 1.01–3.21	LRT *p* = 0.065	No statistically significant improvement in primary-endpoint model fit.
MADIT components + Rotation Score vs. MADIT components	Death without prior therapy	HR 1.10, 95% CI 0.54–2.27	LRT *p* = 0.790	No statistically significant model-fit improvement.
MADIT components + Rotation Score vs. MADIT components	Appropriate ICD shock	HR 2.25, 95% CI 1.21–4.16	LRT *p* = 0.016	Statistically significant improvement in model fit for the secondary appropriate ICD shock endpoint; based on 8 events.
MADIT components + Rotation Score vs. MADIT components	Death without prior shock	HR 0.97, 95% CI 0.48–2.00	LRT *p* = 0.942	No statistically significant model-fit improvement.

Footnotes: Rotation estimates are hazard ratios per 1 SD increase in Rotation Score from cause-specific Cox models. Nested model results are likelihood-ratio test *p* values comparing the MADIT-only model with the corresponding MADIT-plus-Rotation model. MADIT components comprise the VT/VF risk score (0 to 13) and non-arrhythmic death score (−1 to 10). The MADIT ICD benefit score is scaled from 0 to 100. CI, confidence interval; HR, hazard ratio; LRT, likelihood-ratio test; VT/VF, ventricular tachycardia/ventricular fibrillation. The statistically significant comparison for appropriate ICD shock was based on 8 events and should not be interpreted as establishing clinical incremental value, reclassification performance, or clinical utility.

## Data Availability

De-identified data supporting the findings of this study may be available from the corresponding author upon reasonable request, subject to institutional governance, ethics approvals, and data-sharing agreements.

## Supplementary Materials

The following supporting information can be downloaded at: https://www.mdpi.com/article/10.3390/jcm15186985/s1, Table S1. Baseline characteristics of Cohort A and Cohort B. Values are mean ± SD or n/N (%); Table S2. Event counts by prespecified strata; Table S3. Compact cause-specific Cox model outputs. Hazard ratios are shown as HR (95% CI). Rotation Score is expressed per 1 SD increase. Benefit score is per 10-point increase. Warnings indicate sparse-data convergence or separation issues; Table S4. Bootstrap optimism-corrected prediction validation for 2-year appropriate ICD therapy in Cohort A; Table S5. PROFID-aligned Cohort B: NYHA status and Rotation Score as complementary markers of competing risk and arrhythmic risk; Table S6. Schoenfeld residual tests of the proportional-hazards assumption for prespecified Cohort A cause-specific Cox models.

## Abbreviations

ATP	Anti-tachycardia pacing
CRT-D	Cardiac resynchronisation therapy- defibrillator
CI	Confidence interval
CIFs	Cumulative incidence functions
ECG	Electrocardiogram
HR	Hazard ratio
ICD	Implantable cardioverter-defibrillator
LVEF	Left ventricular ejection fraction
MCG	Magnetocardiography
MI	Myocardial infarction
NYHA	New York Heart Association
SD	Standard deviation
SCD	Sudden cardiac death
VCG	Vectorcardiography
VA	Ventricular arrhythmia
VT	Ventricular tachycardia
VF	Ventricular fibrillation
