# Supplementary material for: Magnetocardiographic Rotation Score and Arrhythmic Endpoints in Primary-Prevention ICD Recipients: An Exploratory Substudy of the Magneto-SCD Trial
Source: J Clin Med. 2026 Sep 9;15(18):6985. doi: 10.3390/jcm15186985 (PMC13607692; doi:10.3390/jcm15186985)
Supplement: Supplementary file 1 [file jcm-15-06985-s001.zip › jcm-4466714-supplementary.pdf]

Journal of Clinical Medicine

Table S1. Baseline characteristics of Cohort A and Cohort B. Values are mean ± SD or n/N (%).

Characteristic	Cohort A: primary prevention, N=54	Cohort B: Cohort A + prior MI, N=28
Age, years	63.8 ± 13.2	64.3 ± 12.6
Male sex	45/54 (83.3%)	24/28 (85.7%)
LVEF, %	28.9 ± 11.1	28.3 ± 5.5
ECG QRS duration, ms	116.7 ± 29.6	112.2 ± 27.8
NYHA class ≥2	38/52 (73.1%)	22/27 (81.5%)
Ischaemic aetiology	37/53 (69.8%)	28/28 (100.0%)
Prior myocardial infarction	28/53 (52.8%)	28/28 (100.0%)
Diabetes	19/54 (35.2%)	13/28 (46.4%)
Hypertension	19/54 (35.2%)	10/28 (35.7%)
Chronic Kidney Disease (CKD ≥3)	1/51 (2.0%)	0/26 (0.0%)
CRT-D device	21/54 (38.9%)	8/28 (28.6%)
ACE inhibitor/ARB	11/54 (20.4%)	8/28 (28.6%)
Mineralocorticoid receptor antagonist	31/54 (57.4%)	15/28 (53.6%)
Sacubitril/valsartan	37/54 (68.5%)	17/28 (60.7%)
Beta-blocker	51/54 (94.4%)	27/28 (96.4%)
SGLT2 inhibitor	10/54 (18.5%)	5/28 (17.9%)
Amiodarone	2/54 (3.7%)	1/28 (3.6%)
MCG Rotation Score	451.3 ± 194.2	422.1 ± 161.6
Angular Dynamics	2.9 ± 0.9	2.8 ± 0.7

Footnote: NYHA status was unavailable or unclassifiable in 2 Cohort A participants and 1 Cohort B participant.

Table S2. Event counts by prespecified strata.

Cohort/stratification	Stratum	N	Therapy	DWT	Censored	Shock	DWS	Shock-censored
Cohort A: Rotation Score tertile	T1	18	1	3	14	0	4	14
	T2	18	4	5	9	3	5	10
	T3	18	5	2	11	5	2	11
Cohort A: MADIT VT/VF tertile	T1	18	3	3	12	3	3	12
	T2	18	4	5	9	4	5	9
	T3	18	3	2	13	1	3	14
Cohort A: MADIT non-arrhythmic death tertile	T1	18	3	2	13	3	2	13
	T2	18	3	6	9	2	6	10
	T3	18	4	2	12	3	3	12
Cohort A: MADIT ICD benefit tertile	T1	18	2	4	12	2	4	12
	T2	18	6	4	8	5	5	8
	T3	17	2	2	13	1	2	14
Cohort B: Rotation Score tertile	T1	10	0	2	8	0	2	8
	T2	9	2	4	3	1	4	4
	T3	9	3	1	5	3	1	5
Cohort B: NYHA stratum	NYHA <2	5	1	0	4	1	0	4
	NYHA ≥2	22	4	7	11	3	7	12

Footnote: "Therapy" denotes adjudicated appropriate ICD therapy and "Shock" denotes adjudicated appropriate ICD shock. Cohort B NYHA-stratified rows exclude one participant with missing/unclassifiable NYHA status. Tertile definitions: Cohort A: Rotation Score tertiles were 180.0–323.8, 328.7–504.2, and 523.2–1113.1; MADIT VT/VF score tertiles were 2–6, 6–8, and 8–9; MADIT non-arrhythmic death score tertiles were −1–3, 3–4, and 4–8; and MADIT ICD benefit score tertiles were 11–47, 47–56, and 56–83. Cohort B: Rotation Score tertiles were 180.0–342.7, 348.5–408.7, and 417.7–799.6. Because MADIT scores are integer-valued, tied values at tertile boundaries may appear in adjacent rank-based strata. Cohort B NYHA-stratified rows exclude one participant with missing/unclassifiable NYHA status. Abbreviations: DWT, death without prior therapy; DWS, death without prior shock.

Table S3. Compact cause-specific Cox model outputs. Hazard ratios are shown as HR (95% CI). Rotation Score is expressed per 1 SD increase. Benefit score is per 10-point increase. Warnings indicate sparse-data convergence or separation issues.

Cohort	Endpoint	Model	Term	N	Events	HR (95% CI)	p
A	Appropriate ICD therapy	Rotation Score only	Rotation Score	54	10	1.64 (0.93–2.87)	0.086
A	Death without prior therapy	Rotation Score only	Rotation Score	54	10	1.08 (0.54–2.17)	0.824
A	Appropriate ICD shock	Rotation Score only	Rotation Score	54	8	2.07 (1.15–3.72)	0.015
A	Death without prior shock	Rotation Score only	Rotation Score	54	11	0.95 (0.48–1.90)	0.890
A	Appropriate ICD therapy	MADIT benefit	Benefit score/10	53	10	1.07 (0.76–1.51)	0.688
A	Appropriate ICD therapy	MADIT benefit + Rotation Score	Benefit score/10	53	10	1.10 (0.80–1.50)	0.553
A	Appropriate ICD therapy	MADIT benefit + Rotation Score	Rotation Score	53	10	1.65 (0.93–2.94)	0.090
A	Death without prior therapy	MADIT benefit	Benefit score/10	53	10	0.85 (0.60–1.21)	0.364
A	Death without prior therapy	MADIT benefit + Rotation Score	Benefit score/10	53	10	0.85 (0.60–1.21)	0.372
A	Death without prior therapy	MADIT benefit + Rotation Score	Rotation Score	53	10	1.00 (0.49–2.06)	0.992
A	Appropriate ICD therapy	MADIT components	VT/VF score	54	10	1.08 (0.69–1.69)	0.746
A	Appropriate ICD therapy	MADIT components	Non-arrhythmic death score	54	10	1.35 (0.95–1.92)	0.090
A	Appropriate ICD therapy	MADIT components + Rotation Score	VT/VF score	54	10	1.16 (0.72–1.88)	0.540
A	Appropriate ICD therapy	MADIT components	Non-arrhythmic death score	54	10	1.37 (0.98–1.92)	0.070

		+ Rotation Score					
A	Appropriate ICD therapy	MADIT components + Rotation Score	Rotation Score	54	10	1.80 (1.01–3.21)	0.048
A	Death without prior therapy	MADIT components	VT/VF score	54	10	0.92 (0.62–1.37)	0.671
A	Death without prior therapy	MADIT components	Non-ar-rhythmic death score	54	10	1.13 (0.79–1.61)	0.493
A	Death without prior therapy	MADIT components + Rotation Score	VT/VF score	54	10	0.92 (0.62–1.38)	0.700
A	Death without prior therapy	MADIT components + Rotation Score	Non-ar-rhythmic death score	54	10	1.14 (0.80–1.62)	0.476
A	Death without prior therapy	MADIT components + Rotation Score	Rotation Score	54	10	1.10 (0.54–2.27)	0.787
A	Appropriate shock	MADIT components	VT/VF score	54	8	0.89 (0.56–1.41)	0.619
A	Appropriate ICD shock	MADIT components	Non-ar-rhythmic death score	54	8	1.46 (0.99–2.17)	0.058
A	Appropriate ICD shock	MADIT components + Rotation Score	VT/VF score	54	8	0.97 (0.58–1.62)	0.895
A	Appropriate ICD shock	MADIT components + Rotation Score	Non-ar-rhythmic death score	54	8	1.50 (1.01–2.21)	0.043
A	Appropriate ICD shock	MADIT components + Rotation Score	Rotation Score	54	8	2.25 (1.21–4.16)	0.010
A	Death without prior shock	MADIT components	VT/VF score	54	11	0.95 (0.64–1.40)	0.797

A	Death without prior shock	MADIT components	Non-arrhythmic death score	54	11	1.15 (0.82–1.61)	0.410
A	Death without prior shock	MADIT components + Rotation Score	VT/VF score	54	11	0.95 (0.64–1.41)	0.790
A	Death without prior shock	MADIT components + Rotation Score	Non-arrhythmic death score	54	11	1.15 (0.82–1.61)	0.415
A	Death without prior shock	MADIT components + Rotation Score	Rotation Score	54	11	0.97 (0.48–2.00)	0.943
B	Appropriate ICD therapy	Rotation Score only	Rotation Score	28	5	1.95 (0.89–4.28)	0.097
B	Appropriate ICD therapy	NYHA only	NYHA ≥2	27	5	0.95 (0.11–8.61)	0.966
B	Appropriate ICD therapy	NYHA + Rotation Score	NYHA ≥2	27	5	0.44 (0.04–5.39)	0.523
B	Appropriate ICD therapy	NYHA + Rotation Score	Rotation Score	27	5	2.20 (0.94–5.17)	0.070
B	Death without prior therapy	Rotation Score only	Rotation Score	28	7	0.61 (0.20–1.88)	0.387
B	Death without prior therapy	NYHA only	NYHA ≥2	27	7	Not estimable reliably	—
B	Death without prior therapy	NYHA + Rotation Score	NYHA ≥2	27	7	Not estimable reliably	—
B	Death without prior therapy	NYHA + Rotation	Rotation Score	27	7	0.51 (0.15–1.75)	0.285

Footnote: Cohort A clinical multivariable therapy models were unstable and were omitted from this compact table. In Cohort B death models, all deaths without prior therapy among participants with available NYHA status occurred in NYHA ≥2 patients, producing separation and unstable NYHA coefficient estimates.

Table S4. Bootstrap optimism-corrected prediction validation for 2-year appropriate ICD therapy in Cohort A.

Model	N	Events	Successful bootstrap resamples	Apparent C-index	Optimism-corrected C-index	Corrected calibration slope	Corrected calibration MAE
Rotation Score only	54	10	499/500	0.656	0.633	1.023	0.045
MADIT benefit	53	10	498/500	0.543	0.496	0.122	0.119
MADIT benefit + Rotation Score	53	10	498/500	0.660	0.617	0.777	0.048
MADIT components	54	10	499/500	0.640	0.589	0.782	0.017
MADIT components + Rotation Score	54	10	499/500	0.724	0.657	0.739	0.072
Clinical model	52	10	0/500	Not estimable	Not estimable	Not estimable	Not estimable
Clinical model + Rotation Score	52	10	448/500	0.718	0.582	0.302	0.053

Footnote: Bootstrap validation used 500 requested resamples. C-index is Harrell's C-index. Calibration MAE is the mean absolute difference between observed and predicted 2-year risk across risk groups. Prediction metrics are exploratory because of sparse events and model instability.

Table S5. PROFID-aligned Cohort B: NYHA status and Rotation Score as complementary markers of competing risk and arrhythmic risk.

Cohort B subgroup	N	Appropriate ICD Therapy, full follow-up	Appropriate ICD Shock, full follow-up	Death without therapy, full follow-up	Appropriate ICD Therapy by 2 years	Appropriate ICD Shock by 2 years	Death without therapy by 2 years
NYHA <2	5	1	1	0	0	0	0
NYHA ≥2	22	4	3	7	4	3	4
Low Rotation Score	14	1	0	3	1	0	2
High Rotation Score	14	4	4	4	3	3	2
NYHA <2 + low Rotation Score	4	0	0	0	0	0	0
NYHA <2 + high Rotation Score	1	1	1	0	0	0	0
NYHA ≥2 + low Rotation Score	10	1	0	3	1	0	2
NYHA ≥2 + high Rotation Score	12	3	3	4	3	3	2

Footnote: Rotation Score groups are split at the Cohort B median Rotation Score. One Cohort B participant had missing/unclassifiable NYHA status and is excluded from NYHA-stratified and NYHA-by-Rotation Score rows. Two-year counts are descriptive event counts by 730 days and are not model-based cumulative-incidence estimates.

Table S6. Schoenfeld residual tests of the proportional-hazards assumption for prespecified Cohort A cause-specific Cox models.

Endpoint	Model	Diagnostic	Chi-squared	df	p value
Appropriate ICD therapy	Rotation Score only	Rotation Score	0.377	1	0.539
Death without prior therapy	Rotation Score only	Rotation Score	0.015	1	0.902
Appropriate ICD shock	Rotation Score only	Rotation Score	0.240	1	0.624
Death without prior shock	Rotation Score only	Rotation Score	0.012	1	0.914
Appropriate ICD therapy	MADIT components + Rotation Score	Global test	2.346	3	0.504
Death without prior therapy	MADIT components + Rotation Score	Global test	0.046	3	0.997
Appropriate ICD shock	MADIT components + Rotation Score	Global test	1.719	3	0.633
Death without prior shock	MADIT components + Rotation Score	Global test	0.079	3	0.994

Footnote: Proportional-hazards assumptions were assessed using tests based on scaled Schoenfeld residuals. For Rotation-only models, the term-specific test is identical to the global test and is therefore shown once. For multivariable MADIT-components-plus-Rotation models, the global test is presented. No test provided evidence of a time-varying covariate effect at the conventional 0.05 level. These diagnostics should be interpreted cautiously because of the small number of endpoint events.
